# The Tumor Suppressive Roles and Prognostic Values of STEAP Family Members in Breast Cancer

**DOI:** 10.1155/2020/9578484

**Published:** 2020-08-03

**Authors:** Hua-Tao Wu, Wen-Jia Chen, Ya Xu, Jia-Xin Shen, Wen-Tian Chen, Jing Liu

**Affiliations:** ^1^Department of General Surgery, The First Affiliated Hospital of Shantou University Medical College, Shantou 515041, China; ^2^Changjiang Scholar's Laboratory/Guangdong Provincial Key Laboratory for Diagnosis and Treatment of Breast Cancer, Shantou University Medical College, Shantou 515041, China; ^3^Department of Physiology/Cancer Research Center, Shantou University Medical College, Shantou 515041, China; ^4^Department of Hematology, The First Affiliated Hospital of Shantou University Medical College, Shantou 515041, China

## Abstract

**Objective:**

To investigate the expression patterns and prognostic values of STEAP family members in the occurrence and development of breast cancer.

**Materials and Methods:**

The Human Protein Atlas was used to analyze the expression level of STEAPs in human normal tissues and malignant tumors. ONCOMINE datasets were analyzed for the comparison of the STEAPs levels between malignant cancers and corresponding normal tissues. Kaplan-Meier plotter was used to analyze the prognostic value of STEAPs in breast cancer patients.

**Results:**

STEAPs were widely distributed in human normal tissues with diverse levels. Normally, it is predicted that STEAP1 and STEAP2 were involved in the mineral absorption process, while STEAP3 participated in the TP53 signaling pathway and iron apoptosis. The results from ONCOMINE showed downregulation of STEAP1, STEAP2, and STEAP4 in breast cancers. Survival analysis revealed that breast cancer patients with high levels of STEAP1, STEAP2, and STEAP4 had a good prognosis, while those with low expression had high overall mortality.

**Conclusion:**

STEAP1, STEAP2, and STEAP4 are predicted to be the potential prognostic biomarkers for breast cancer patients, providing novel therapeutic strategies for them.

## 1. Introduction

Breast cancer (BC), the most common female carcinoma, is the second leading cause of cancer-related death in females worldwide [1]. According to the latest breast cancer statistics in 2019, approximately 316,700 new cases of BC among American women will be diagnosed, and 41,760 women will die from BC [1]. Currently, the expression of reported biomarkers, such as estrogen receptor (ER), progesterone receptor (PR), and human epidermal growth factor receptor 2 (HER2), is used to subtype this disease for further targeted therapy and precision medicine [2]. And more efforts have been performed to explore other biomarkers and exhibited their potential usefulness in predicting therapeutic efficacy and tumor recurrence [3]. Although survival advantages are achieved by surgical resection, combined with radiotherapy, chemotherapy, and/or targeted therapy for special patients with early-stage BC, the locally advanced or metastasis diseases predict poor survival outcomes of a median of 2-3 years period [4]. Therefore, a great challenge is still present to the researchers and doctors for accurate diagnosis and prognosis evaluation.

The human six-transmembrane epithelial antigens of prostate (STEAP) family is a kind of cell surface membrane protein with a similar structure, 6 transmembrane domain, and intracellular amino and carboxyl terminals, including four members, namely, STEAP1, STEAP2, STEAP3, and STEAP4 [5]. Normally, the members of the STEAP family conduct their physiological functions as oxidoreductases, participating in the absorption and reduction of iron and copper [6, 7]. So as expected, the STEAPs are involved in intercellular conduction, oxidative stress, inflammation, cell growth, and differentiation [8]. Previous evidence reveals that STEAP1 is aberrantly high expressed in prostate cancer and predicted as a prostate-specific cell-surface antigen [9]. The subsequent identification of other STEAPs promoted the investigation of their normal and pathological function in different diseases, especially in cancers [10–14]. Recently, Sikkeland et al. systematically reviewed the expression levels of STEAPs in normal tissues and disease states and reported the diverse role of STEAPs in normal and different pathological tissues, revealing that only STEAP3 was reported to be highly expressed in the mammary gland [15]. Both of the prostate and breast cancer are sexual hormone-related cancers [16]; however, the research of the effect of the prostate-specific cell-surface antigen on breast cancer is limited. So this study focused on the diverse expression patterns and prognostic values of STEAPs in breast cancer, to explore their potential values as new biomarkers or therapeutic targets for the diagnosis and precision therapy of breast cancer patients.

## 2. Materials and Methods

### 2.1. The Expression Levels of STEAPs in Normal Tissues and Cancers

The Human Protein Atlas database (https://www.proteinatlas.org/) was used to obtain the expression level of STEAPs at the protein level and mRNA level in normal tissues and different cancers [17]. RNA expression was analyzed in Consensus Normalized eXpression (NX) levels, combining the data from three transcriptomic datasets (HPA, GTEx, and FANTOM5), using the internal normalization pipeline. The RNA expression levels were ranked as four groups: not detected (<1 NX), low expression (≥1 NX and <15 NX), medium expression (≥15 NX and <30 NX), and high expression (≥30NX). The protein levels were also divided into four groups: negative (-), low expression (+), medium expression (++), and high expression (+++).

### 2.2. KEGG Pathway Analysis

Kyoto Encyclopedia of Genes and Genomes (KEGG) database (https://www.kegg.jp/kegg/) was applied to explore the related signal pathways of STEAPs, based on the integration of genomic, chemical, and system functional information [18].

### 2.3. ONCOMINE Analysis

The mRNA levels of different STEAPs in different cancers were analyzed by ONCOMINE datasets (https://www.oncomine.org/), an online cancer microarray database [19]. The compared datasets between the clinical specimens of cancer and normal control were analyzed using Students' *t*-test. And the cutoff of *p* value was set as 1*e* − 4, and fold change was defined as 2. Typical figures were also used to predict the significant correlation in different research.

### 2.4. Kaplan-Meier Plotter

Kaplan-Meier plotter (http://kmplot.com/analysis/), a database of gene expression and clinical data [20], was assessed for the prognostic value of STEAPs in mRNA and protein levels. The patients' samples were divided into high and low expression groups, according to STEAP expression levels. The number-at-risk was indicated below the Kaplan-Meier plot.

## 3. Results

### 3.1. The Expression of STEAP Family Members in Normal Tissues

Based on the analysis of the Human Protein Atlas database (Table 1), it is found that in normal tissues, STEAP1 protein was highly expressed in the lung, moderately expressed in the cerebral cortex, prostate, and testis, and not detected in the breast or other organs. The mRNA level of STEAP1 was highest in prostate tissues, medium expressed in breast, and detected in almost all detected tissues. Oppositely, STEAP2 and STEAP3 proteins can be detected in most organs in medium or high expression levels. However, the protein level of STEAP4 was not examined yet.

### 3.2. The Expression of STEAP Family Members in Different Types of Malignant Tumors

As shown in Table 2, the mRNA level of STEAP1 was low in different types of malignant tumors except for prostate cancer, and the protein level of STEAP1 was medium expressed in the tissues of lung cancers, low or not detected in other types of cancers. Interestingly, the expression of STEAP2 and STEAP3 is similar in different types of malignant tumors, with high or medium levels. The protein level of STEAP4 was also not tested yet.

### 3.3. The Expression Pattern of STEAPs in Breast Cancer and Normal Tissues

**Figure 1:**
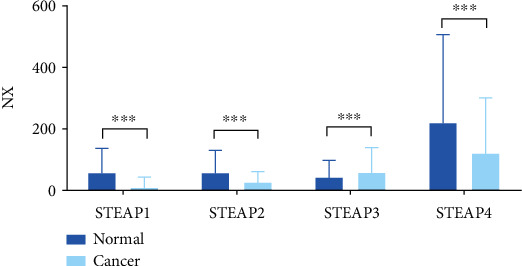
The relative RNA levels of STEAP expression in breast/breast cancers. ∗∗∗ means *p* < 0.001.

The relative RNA expression of STEAPs in breast tissues and breast cancer was analyzed accordingly and found that STEAP1/2/4 were highly expressed in normal mammary glands compared with that in breast cancer tissues (Figure 1).

**Figure 2:**
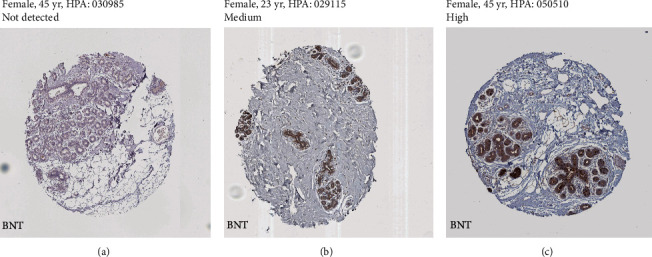
The representative images of STEAP expression in normal breast tissues. (a) STEAP1. (b) STEAP2. (c) STEAP3. Abbreviation: BNT: breast normal tissues.

In normal breast tissues, the protein level of STEAP1 was not detected in glandular cells, whereas medium-expressed STEAP2 and high-expressed STEAP3 were detected in protein levels in breast glandular cells (Figure 2).

**Figure 3:**
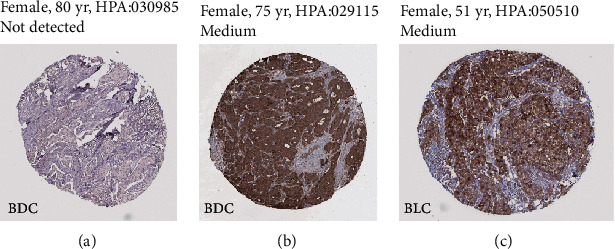
The representative images of STEAP expression in breast cancers. (a) STEAP1. (b) STEAP2. (c) STEAP3. Abbreviation: BDC: breast ductal carcinoma; BLC: breast lobular carcinoma; HPA: Human Protein Atlas.

For breast cancer, the expression of STEAP1 was not detected in protein levels, whereas the protein levels of STEAP2 and STEAP3 were medium expressed in breast cancer tissues (Figure 3).

### 3.4. The Comparison of the Expression of STEAPs in Breast Cancer and Normal Tissues

**Figure 4:**
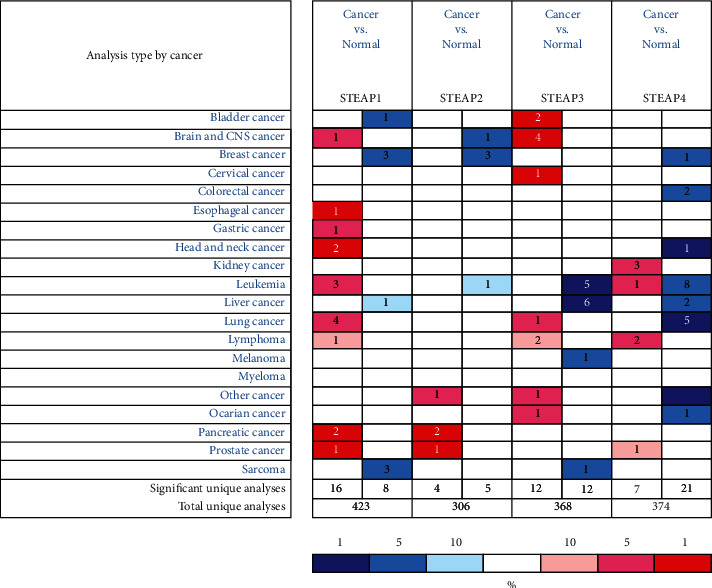
The comparison of the expression of STEAPs in different types of cancers and corresponding normal tissues. This figure revealed the numbers of datasets with statistically significant mRNA over- (red) or down- (blue) expression of STEAPs. The *p* value threshold is 1*e* − 4, and the cell color is decided by the best gene rank percentile for the analyses in each cell.

After comparing the expression of STEAPs in different types of malignant cancers with their corresponding normal tissues, it is shown that the expression pattern of these four enzymes is different in types of cancers, predicting their diverse role in the occurrence and development of different types of malignant tumors (Figure 4).

**Figure 5:**
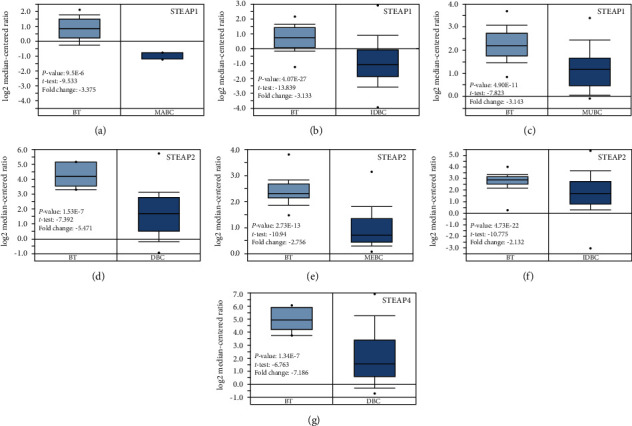
The expression of STEAPs in breast cancers. Box plots were obtained from ONCOMINE comparing the expression of STEAPs in normal and BC tissues. The *p* value was set up at 1*e* − 4, and fold change was defined as 2. (a–c) Comparison of STEAP1 expression. (d–f) Comparison of STEAP2 expression. (g) Comparison of STEAP4. Abbreviation: BT: breast tissue; MABC: male breast carcinoma; IDBC: invasion ductal breast carcinoma; MUBC: mucinous breast carcinoma; DBC: ductal breast carcinoma; MEBC: medullary breast carcinoma.

For breast cancer, three datasets showed lower expression of STEAP1 (Figures 5(a)–5(c)) and STEAP2 (Figures 5(d)–5(f)) in tissues of breast cancer, compared with normal breast tissues [21, 22]. The mRNA level of STEAP4 was also decreased in tissues of ductal breast carcinoma, compared with normal tissues, with -7.186 fold change (*p* = 1.34*e* − 7) [22] (Figure 5(g)). However, no significant aberrant expression of STEAP3 was found in breast cancer tissues based on the analysis of ONCOMINE database.

### 3.5. The Prognostic Value of Different STEAPs in Breast Cancer

**Figure 6:**
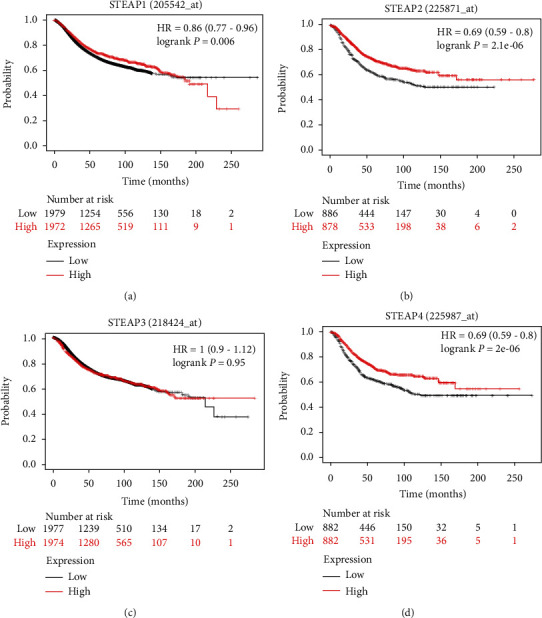
The prognostic evaluation of STEAPs in patients with breast cancers. (a) STEAP1. (b) STEAP2. (c) STEAP3. (d) STEAP4.

A positive relationship between the STEAP1 mRNA level and the overall survival (OS) in breast cancer was found (*p* = 0.006). BC patients with a high level of STEAP1 were predicted to have a long survival period (Figure 6(a)). It was also revealed that the expression of STEAP2 and STEAP3 mRNA was also potential good predictors for BC patients. The BC patients with high expression of STEAP2 or STEAP3 had a long survival period, with the same hazard ratio (HR) = 0.69 (0.59-0.8) and different *p* value = 2.1*e* − 6 and 2*e* − 6, respectively (Figures 6(b) and 6(d)). However, the expression of STEAP3 did not affect the OS of BC patients (Figure 6(c)).

## 4. Discussion

**Figure 7:**
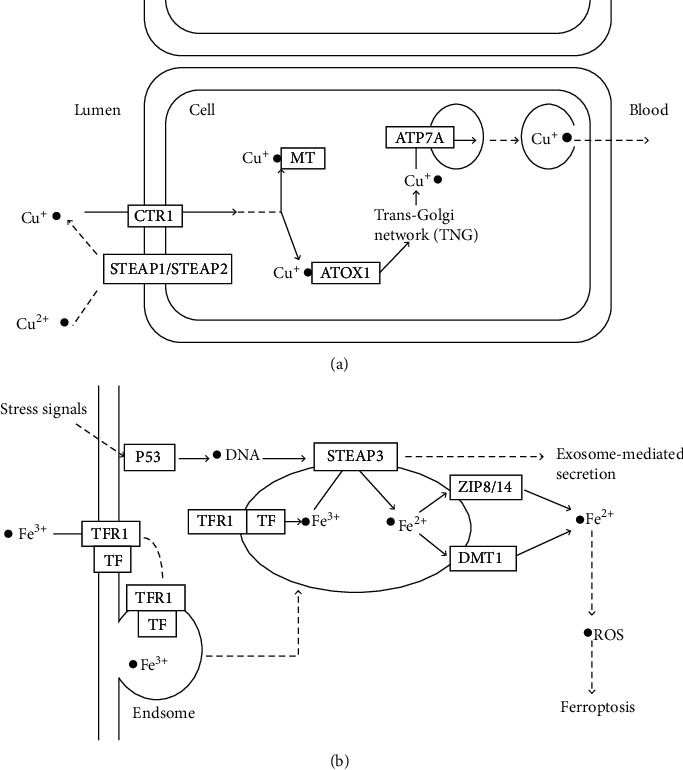
The STEAPs involved pathways based on KEGG analysis. (a) STEAP1 and STEAP2 are predicted to be involved in mineral absorption. (b) STEAP3 is predicted to participate in the processes of TP53-regulating transcription of cell death genes and ferroptosis. Abbreviations: CTR1: copper transporter member 1; MT: metallothionein-1; ATP7P: ATPase copper transporting alpha; ATOX1: antioxidant 1 copper chaperone; TFR1: transferrin receptor; TF: transferrin; ZIP8: zinc transporter member 8; DMT1: ferrous ion membrane transport protein DMT1; ROS: ROS protooncogene 1.

Currently, the investigation of gene expression in malignant tumors provides tremendous prediction and prognosis information for diagnoses and therapies of patients [23]. The members of the STEAP family are relatively new-discovered proteins [9, 24–27]; correspondingly, the research also relatively limited on STEAPs. As the STEAP family is widely expressed in normal human tissues, their important role was confirmed previously in normal pathological processes through mineral absorption, and TP53-regulating transcription of cell death genes and ferroptosis (Figure 7). However, the role and underlying molecular mechanism of STEAPs in oncogenesis and development of breast cancer need further investigation. Although the aberrant expression of STEAPs has been reported in multiple cancers, the expression patterns and prognostic values of STEAPs in breast cancer are still unclear. This study expanded the knowledge of STEAPs in breast cancer and revealed the potentials of STEAPs for the therapy targets and prognostic biomarkers in breast cancer.

It was proved that the expression of STEAP1 in prostate cancer is significantly increased, and silencing STEAP1 expression can inhibit the proliferation of prostate cancer cells and promote apoptosis [10]. Furthermore, the upregulation of STEAP1 has been also detected in lung, gastric, colorectal, renal, and bladder cancer [28–31]. Maia et al. demonstrated that STEAP1 is overexpressed in human breast cancer cases [32]. On the contrary, Xie et al. found the expression of STEAP1 in breast cancer is decreased and related to lymph node metastasis, cell differentiation, and histological grade. Studies have shown that the downregulation of STEAP1 expression in breast cancer enhances the invasion and migration of cells and increases the expression of EMT-related biomarkers [33]. In the current study, it is found that the expression of STEAP1 in breast cancer was lower than that in normal breast epithelium. Survival analysis revealed that higher expression of STEAP1 correlated with a better outcome.

It was reported that STEAP2 was significantly overexpressed in prostate cancer, and the overexpression of STEAP2 promoted the proliferation, migration, and invasion of tumor cells [12, 34]. Wang et al. found that STEAP2 may influence the progression of prostate cancer by activating the ERK signaling pathway [35]. Besides, STEAP2 was also highly expressed in colorectal cancer and drives the excessive proliferation of colon tumor cells [36]. Surprisingly, low expression of STEAP2 was detected in breast cancer previously [37], consistent with the findings in this study. And suppression of the STEAP2 level can promote cell proliferation and invasion by inducing EMT and activating the PI3K/AKT signaling pathway [37]. Additionally, the expression of STEAP2 also was evaluated to be a potential good predictor for patients with breast cancer.

Kim et al. proved that STEAP1 may form homologous trimer or form heterotrimer with STEAP2 by transferring an electron through the heme group, reducing Fe^3+^ to Fe^2+^ and Cu^2+^ to Cu^+^ [38]. Ramos et al. demonstrated that STEAP2 on the cell surface may interact with ceruloplasmin to form keratin and participate in the uptake of copper [39]. As minerals are essential nutrients to sustain life, their absorption via passive or active transport systems is important and STEAP is supposed to be one of the special transport proteins. STEAP1-4 are six-transmembrane protein structure; both ends of the intracellular hydrophilic amino and carboxyl, amino on paleontology and bacteria F420H2: NADP+ oxidoreductase (FNO) sample structure domain, as iron and copper reduction of electron donor, and its prediction combined with at least one film in heme groups, may play a role in the uptake of iron and copper [6, 7, 40, 41]. The current study also predicted the participation of STEAP1 and STEAP2 in the process of mineral absorption through converting Cu^2+^ to Cu^+^ and promoting copper absorption, which may be the underlying mechanism that STEAPs are involved in the development of breast cancers.

Recently, studies on STEAP3 have emerged, predicting the important role of STEAP3 in cancers. Machlenkin et al. reported that STEAP3 was highly expressed in prostate cancer [13]. Subsequently, increased expression of STEAP3 was proved in a variety of tumor tissues, including breast cancer [42–47]. The activation of p53 is induced by a variety of stress signals, including DNA damage, oxidative stress, and activated oncogenes. It is verified that STEAP3 is the direct target gene of TP53, and TP53 can affect the fate of cells through expelling proteins by upregulation of STEAP3 [48]. As showed in Figure 7(b), the exosome-mediated secretion may be regulated by the TP53/STEAP3 pathway. On the other hand, ferroptosis is a regulatory form of cell death, caused by accumulated iron and lipid peroxidation produce reactive oxygen species (ROS), involved in series of physiological and pathological processes, such as cancer cell death, neurodegenerative diseases, tissue injury, and acute renal failure [49, 50]. Song et al. illustrated that FTH1 (ferritin, heavy polypeptide 1), an enzyme, inhibits ferroptosis by binding to Fe^2+^ and STEAP3, converting iron from Fe^3+^ to Fe^2+^ [51]. However, the current results did not show the relationship between STEAP3 levels and the survival of BC patients.

The expression of STEAP4 is also upregulated in prostate cancer, and its oncogenic role in prostate cancer is proved by several studies [14, 52, 53]. The increased expression of STEAP4 is also found in colorectal cancer [54], hepatocellular carcinoma [55], and breast cancer [56]. Interestingly, Yan et al. demonstrated that STEAP4 was decreased in bladder cancer, and the competition between STEAP4 and CircPICALM combined with miR-1265 will affect the EMT process in bladder cancer cells [57]. This study also found the downexpression of STEAP4 in breast cancer, and the expression of STEAP4 is related to the prognosis of breast cancer. The high expression of STEAP4 in breast cancer patients is often accompanied by a long survival period.

Currently, STEAP1B, sharing 88% amino acids with STEAP1, is assigned to the STEAP protein family [8]. However, STEAP1B did not contain a classical six-transmembrane structure and the research on STEAP1B is rarely limited. Even the current tools are not available to investigate the expression and prognostic value of STEAP1B.

## 5. Conclusions

In conclusion, through analyzing multiple databases, it is suggested that among STEAP family members, STEAP1, STEAP2, and STEAP4 have low levels in patients with breast cancer. Moreover, STEAP1, STEAP2, and STEAP4 are related to the prognosis of breast cancer patients, providing an important theoretical basis and clinical guidance for the development of therapeutic targets and drugs for breast cancer patients.

## Figures and Tables

**Table 1:** The expression levels of STEAP family members in human normal tissues.

Organs	STEAP family members							
	STEAP1		STEAP2		STEAP3		STEAP4	
	RNA^∗^	Protein	RNA	Protein	RNA	Protein	RNA	Protein
Cerebral cortex	+	++	++	++	+	-		N.E.^△^
Thyroid gland	+	-	+	++	+	++	+++	N.E.
Lung	+	+++	+	++	+	++	++	N.E.
Esophagus	+	-	+	++	+	++	++	N.E.
Stomach	+	-	+	++	+	++	+	N.E.
Colon/rectum	+	-	+	++	+	++	+	N.E.
Liver	++	-	+	++	+++	++	+++	N.E.
Pancreas	+	-	+	++	+	+	+	N.E.
Kidney	+	-	+	++	+	++	+	N.E.
Urinary bladder	+	-	+	++	+	++	++	N.E.
Testis	+	++	+	++	+	++	++	N.E.
Prostate	+++	++	+++	++	+	++	++	N.E.
Ovary	+	-	++	+	+	++	++	N.E.
Endometrium	+	-	+	++	+	++	+	N.E.
Cervix uterine	+	-	+	++	+	++	+	N.E.
Breast	++	-	+	++	+	++	+++	N.E.
Skin	+	-	+	++	+	+	+	N.E.
Lymph node	+	-	+	++	+	++	+++	N.E.
Bone marrow	-	-	+	++	+	+	+	N.E.

^∗^RNA and protein expression: not detected (-); low levels (+); medium levels (++); high levels (+++). ^△^N.E. means not examined.

**Table 2:** The expression levels of STEAP family members in different types of malignant tumors.

Malignant tumors	STEAP family members							
	STEAP1		STEAP2		STEAP3		STEAP4	
	RNA^∗^	Protein	RNA	Protein	RNA	Protein	RNA	Protein
Glioma	+	-	-	++	++	+	-	N.E.^△^
Thyroid cancer	-	-	-	+++	+	-	+	N.E.
Lung cancer	+	++	+	+++	++	++	+	N.E.
Colorectal cancer	+	-	+	+++	+	+++	-	N.E.
Head and neck cancer	+	-	+	+++	+	+	+	N.E.
Stomach cancer	+	+	+	+++	+	++	+	N.E.
Liver cancer	+	-	-	+++	++	+++	-	N.E.
Pancreatic cancer	+	-	+	+++	+	+	+	N.E.
Renal cancer	+	-	+	+++	+	+++	+	N.E.
Urothelial cancer	+	-	+	++	+	+++	-	N.E.
Prostate cancer	+++	+	+++	+++	+	+++	+++	N.E.
Testis cancer	+	-	-	+++	+	++	-	N.E.
Breast cancer	+	-	+	++	+	++	+	N.E.
Cervical cancer	+	-	+	+++	+	+++	+	N.E.
Endometrial cancer	+	-	+	+++	+	+++	-	N.E.
Ovarian cancer	+	-	+	+++	+	+	-	N.E.
Melanoma	+	-	-	++	+	++	-	N.E.
Skin cancer	-	-	-	+	-	+	-	N.E.
Lymphoma	-	-	-	+	-	-	-	N.E.

^∗^RNA and protein expression: not detected (-); low levels (+); medium levels (++); high levels (+++). ^△^N.E. means not examined.

## Data Availability

The data used in this study are available upon request from the corresponding author.
